# Systematic comparison of tissue fixation with alternative fixatives to conventional tissue fixation with buffered formalin in a xenograft-based model

**DOI:** 10.1007/s00428-012-1248-5

**Published:** 2012-07-20

**Authors:** Thorben Nietner, Tiantom Jarutat, Alfred Mertens

**Affiliations:** 1 University of Hohenheim, 70593 Stuttgart, Germany; 2 Roche Diagnostics GmbH, Nonnenwald 2, 82377 Penzberg, Germany

**Keywords:** Formalin fixation, Immunohistochemistry, AFA, HOPE®, PAXgene®, Ultrasound

## Abstract

In our study we systematically compared the alternative fixatives acidified formal alcohol (AFA), PAXgene®, HOPE®, and combinations of AFA or formalin with ultrasound treatment to standard (buffered) formalin fixation. We examined general morphology and detectability of protein structures by immunohistochemistry of the membrane receptors epidermal growth factor receptor (EGFR), insulin-like growth factor 1 receptor (IGF-1R), and phosphorylated human epidermal growth factor receptor 2 (phospho-HER2). In order to allow for stringent comparability of different fixation techniques, we used matched mouse xenograft tumor samples from three different human cancer cell lines (colon, ovarian, and non-small cell lung cancer), either fixed conventionally with formalin or an alternative fixative. Tissue morphology after fixation with AFA and PAXgene® was comparable to formalin-fixed paraffin-embedded tissue (FFPET) morphology. Ultrasound fixations resulted in slightly inferior morphology and HOPE® fixation preserved morphology only poorly compared to FFPET in this system. None of the tested alternative fixatives enabled immunohistochemical detectability of all three targets in the same manner as FFPET. Pronounced staining was possible for EGFR and IGF-1R with all alternative fixatives but HOPE®, and phospho-HER2 staining was only noteworthy with formalin-ultrasound-fixed tissue. Therefore, the use of alternative fixatives comes with the need for careful validation of obtained IHC results individually for each target.

**Electronic supplementary material:**

The online version of this article (doi:10.1007/s00428-012-1248-5) contains supplementary material, which is available to authorized users.

## Introduction

Antibody-based therapeutics and targeted therapies in oncology have achieved great importance in recent years. With the ongoing development of personalized healthcare, pathologists are in need of specific diagnostics, often based on protein detection in patients’ tissue. Tissue diagnostics in biomedical research and routine practice are either performed with fresh frozen tissue or with formalin-fixed tissue, which has been used in histopathology for decades, firstly mentioned by Blum in 1893 [1, 2]. The advantages of formalin are firstly the antibacterial and antiviral effect, which protects laboratory personnel from infections and secondly the complete fixation of the tissue due to the chemical properties of formaldehyde [3]. However, formalin is toxic and its cross-linking effect on tissue structures often makes specific bioanalysis of proteins and nucleic acids difficult [4–9]. Therefore, powerful fixation techniques are needed which can produce highly conserved tissues from which an analysis for every target is possible.

In the past years, many alternative fixation studies have been carried out, most of them dealing with one novel or optimized fixative in comparison to standard formalin, but only a few studies on many different fixatives were conducted. Comparative studies between formalin and an alternative fixative for example, were about Streck’s tissue fixative [9], Bouin’s fixative [10], methacarn [11], ethanol [5], HOPE® [12–18], and UMFIX [8, 19, 20]. Broad-ranging studies on various fixatives for example have been from Prento and Lyon [21] in 1997 (Histochoice, Kryofix, Mirsky’s fixative, NoTox, Omnifix II, Streck’s Tissue Fixative/Tissue-Tek, Clarke’s ethanol-acetic acid, and ethanol) and from Atkins et al. [7] in 2004 (Bouin’s fixative, AFA, PreFer, and Pen-fix).

The aim of this study was to compare standard buffered formalin fixation (3.9 % formaldehyde, *w*/*v*) to fixation with acidified formal alcohol (AFA), which is widely used in France, with HOPE® fixation [12–18], with the recently introduced non-cross-linking fixative PAXgene® [22, 23] and with combinations of AFA and formalin with ultrasound treatment, since ultrasound-accelerated tissue fixation has been reported for formalin fixation previously [24–28]. In order to allow for stringent comparability of the different fixation techniques matched mouse xenograft tumor samples were used. The samples were removed in the exact same manner with minimized ischemia ranging at most to 60 s and standardized fixation parameters such as duration or temperature were applied.

On the other hand, the study was aligned to routine histopathology and downstream workflows in pathological laboratories as much as possible. Therefore, alternative fixations were coupled to the usual dehydration process, subsequent paraffin embedding and fully automated immunohistochemistry on the BenchMark XT instrument (Ventana Medical Systems; Tucson, USA), which at the same time enabled maximized standardization. Comparison of alternative fixatives was also oriented to immunohistochemical targets which have significant impact on patient care as well as to exploratory tissue biomarkers. Immunohistochemistry was applied for epidermal growth factor receptor (EGFR) as an established target in cancer diagnostics, insulin-like growth factor 1 receptor (IGF-1R) as an exploratory marker and phosphorylated human epidermal growth factor receptor 2 (phospho-HER2) as a signal transduction marker in order to test detectability of phosphorylated antigens.

## Material and methods

**Fig. 1:**
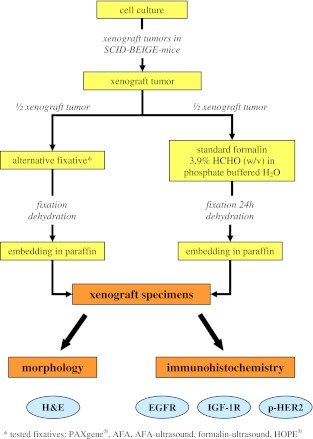
Experimental setup

In the study, the alternative fixatives AFA, PAXgene®, HOPE®, and combinations of AFA or formalin with ultrasound treatment were compared to standard (buffered) formalin fixation. For this comparison tumor samples obtained from mouse xenograft tissue models of the human cancer cell lines COLO-205, OVCAR-5, and NCI-H322M were used. For every alternative fixative at least five xenografts of every cell line from five different mice were generated. In order to systematically compare all fixatives with formalin, one half of every tumor underwent fixation in standard formalin and the other half of this tumor was fixed in one of the alternative fixatives (Fig. 1). For comparison of alternative fixation with formalin fixation both H&E staining and IHC staining of the membrane receptors EGFR, IGF-1R, and phospho-HER2 were performed (Fig. 1).

For detailed methodology of the study and fixation protocols please refer to Supplementary file 1. Staining protocols of IHC targets on the BenchMark XT instrument (Ventana Medical Systems; Tucson, USA) are included in Supplementary file 2.

## Results

In an initial screen, we selected the best fixation conditions for fixation of xenografts with AFA, AFA-ultrasound, formalin-ultrasound, PAXgene®, and HOPE® by evaluation of tissue preservation/tissue morphology. It was assumed that a fixation which allows good preservation of morphological details would be beneficial for IHC analyses.

### Morphology

**Fig. 2:**
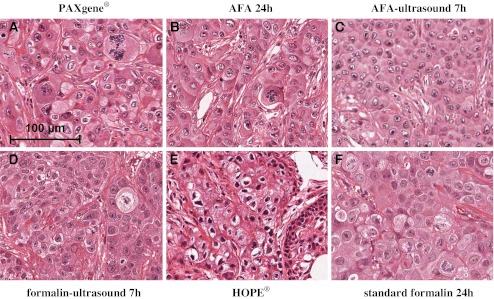
H&E staining of NCI-H322M xenografts **a** PAXgene®, **b** AFA 24 h, **c** AFA-ultrasound 7 h, **d** formalin-ultrasound 7 h, **e** HOPE®, **f** standard formalin 24 h

H&E-stained sections of NCI-H322M xenograft tissue of all tested fixatives are shown in Fig. 2. Preservation of xenograft tissue structures was comparable to standard formalin fixation after fixation with AFA for 24 h, PAXgene®, and ultrasound fixation with AFA and formalin (both for 7 h). Tissue structures were well preserved for the abovementioned fixatives and, in the cases of AFA 24 h, PAXgene®, and AFA-ultrasound fixation, more nuclear details like nucleoli and mitoses were detectable (Fig. 2a–c). HOPE® fixation however, led to decreased morphological detail compared to standard formalin fixation, in which many nuclei developed a characteristic condensed and polygonal shape. Furthermore, the cytoplasm of some cells was shrunken to a considerably larger extent than with the standard FFPET method and typical tissue architecture was partially destroyed after fixation with HOPE®, visible in artificial fissures (Fig. 2e).

### Immunohistochemistry

#### Optimization of immunohistochemical staining methods

Immunohistochemical assay parameters like deparaffinization and antigen retrieval times were optimized individually for each fixative other than standard formalin for maximal IHC signal over background when analyzing xenografts from COLO-205 cells. The resulting modified protocols were then applied accordingly to the other xenograft models. In summary, for fixation with AFA, PAXgene®, and AFA-ultrasound, manual deparaffinization led to better staining results (intensity and quantity) than the automatic deparaffinization using the BenchMark XT for all tested IHC targets. In most of the cases, antigen retrieval times were shorter or of the same duration as for the use of standard formalin-fixed tissue. For the detailed methodology and results refer to Supplementary file 3.

#### Immunohistochemical staining of EGFR, IGF-1R, and phospho-HER2

##### EGFR

**Fig. 3:**
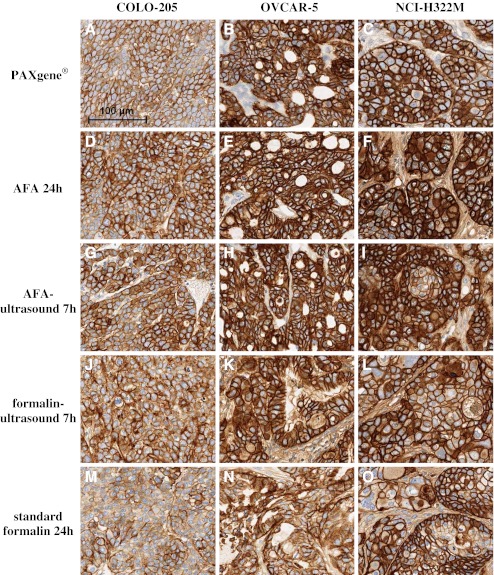
IHC staining with anti-EGFR of different xenograft tissues fixed with alternative fixation methods and standard formalin. All pictures are shown in the same scale (*100-μm scale bar picture top left*)

Staining of EGFR obtained from fixation with AFA, AFA-ultrasound, and PAXgene® resulted in very intense staining with more cells stained than in standard formalin-fixed tissue. For AFA and AFA-ultrasound the EGFR staining was comparable for all three xenograft models (COLO-205, OVCAR-5, and NCI-H322M) and, in comparison to standard formalin, more intensive in all cases with a good counterstaining and detailed nuclear morphology (Fig. 3d–i). PAXgene® fixation led to better staining results of EGFR for all xenograft models (Fig. 3a–c); whereas the difference to standard formalin was smallest for COLO-205 xenografts. Fixation with formalin-ultrasound led to slightly stronger EGFR staining intensity with a few more cells stained in OVCAR-5 and NCI-H322M xenografts and to the same EGFR staining intensity with more cells stained (∼5–10 %) for COLO-205 xenografts compared to standard formalin fixation (Fig. 3j–o). The detection of EGFR was not possible for tissue that was fixed with HOPE® due to unspecific staining, which also occurred when performing a negative control with an IgG control antibody.

##### IGF-1R

**Fig. 4:**
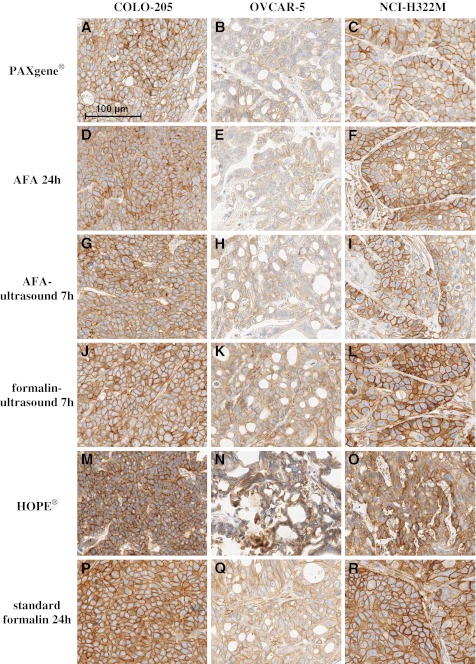
IHC staining with anti-IGF-1R of different xenograft tissues fixed with alternative fixation methods and standard formalin. All pictures are shown in the same scale (*100-μm scale bar picture top left*)

Detection of IGF-1R was possible with all alternatively fixed tissues. The best results, which were nearly comparable to the IGF-1R staining of standard formalin-fixed tissue, were achieved with COLO-205 xenografts fixed with AFA, AFA-ultrasound, and PAXgene®. However, for NCI-H322M and OVCAR-5 xenografts, quantity of IGF-1R staining was less for tissue fixed with AFA, AFA-ultrasound, and PAXgene® than for standard formalin-fixed tissue (Fig. 4a–i and p–r). Formalin-ultrasound-fixed tissue showed an IGF-1R staining which was comparable to staining results of standard formalin-fixed tissue (Fig. 4j–l and p–r). HOPE®-fixed tissue showed a more intense IGF-1R staining (Fig. 4m) with fewer cells stained than the standard formalin-fixed tissue for all three xenograft models. Nevertheless, HOPE®-fixed OVCAR-5 and NCI-H322M xenografts (Fig. 4, N+O) showed cells with condensed shape and shrunken cytoplasm, which made histopathological evaluation difficult.

##### Phospho-HER2

**Fig. 5:**
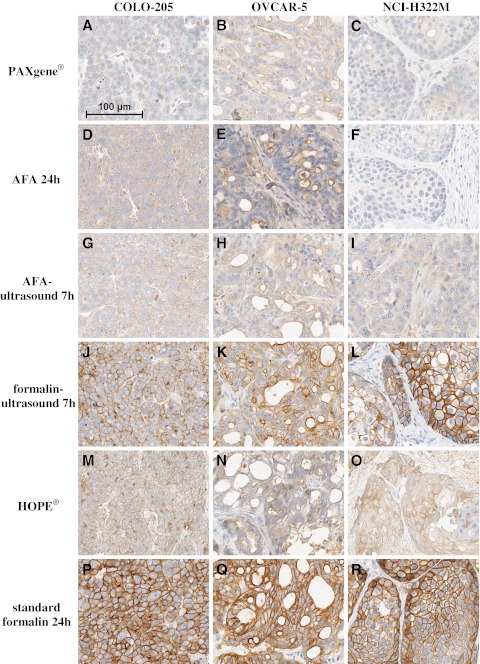
IHC staining with anti-phospho-HER2 of different xenograft tissues fixed with alternative fixation methods and standard formalin. All pictures are shown in the same scale (*100-μm scale bar picture top left*)

The immunohistochemistry of p-HER2 only led to noteworthy staining with formalin-ultrasound-fixed tissue. p-HER2 staining intensity in formalin-ultrasound-fixed tissue (Fig. 5j–l) was comparable to staining intensity of standard formalin-fixed tissue (Fig. 5p–r), whereas the staining quantity in all three xenograft models over all the tumors was less for formalin-ultrasound-fixed tissue. HOPE® fixation led to a weak membrane staining of p-HER2 and to an unspecific cytoplasmic staining for all three xenograft models (Fig. 5m–o). HOPE®-fixed OVCAR-5 xenografts (Fig. 5n) had a decreased morphology which did not allow a histopathological evaluation for p-HER2 staining. No significant staining of p-HER2 could be observed after fixation with AFA, AFA-ultrasound, and PAXgene® (Fig. 5a–i).

All results of the study are summarized in Table 1, showing the results for morphology and the different immunohistochemical targets. Evaluation of IHC experiments with complete scoring is included in Supplementary file 4.

**Table 1:**
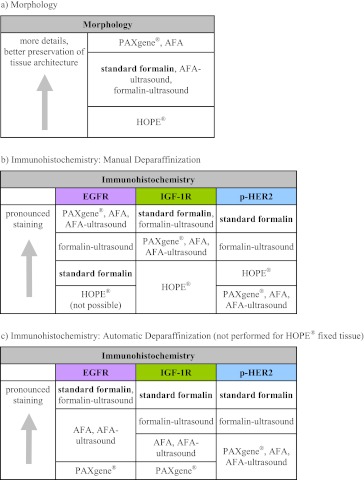
Summary of results

## Discussion

In the present study we demonstrate that careful validation of the usage of alternative fixatives has to be done as the impact on the biomarker results obtained by IHC in relation to standard formalin fixation is significant. A comparison of fixation with formalin, AFA, PAXgene®, HOPE®, and ultrasound accelerated fixation with formalin and AFA was performed on the basis of tissue morphology and immunohistochemical detection of three membrane receptors (EGFR, IGF-1R, and p-HER2). For each fixative five mouse xenograft tumors of three different human cancer cell line models from five different mice have been analyzed. The use of xenograft tissue enabled a direct comparison with human cellular background in a consistency (controlled ischemia, controlled cut halves and infiltration of the fixative to small sample sizes) which would not be achievable with diagnostic human tissue (as biopsies would be too valuable). To our knowledge, this is the first study on alternative fixation with AFA-ultrasound compared to fixation with buffered formalin in a systematic model system.

### Morphology of alternatively fixed tissue

This study clearly showed that the morphology of xenograft tissue was well preserved after fixation with AFA for 24 h, AFA-ultrasound for 7 h, PAXgene® (complete fixation duration 5 h), and formalin-ultrasound for 7 h. The detailed nuclear morphology after fixation with AFA is consistent to the results of Foster et al. [3] who describe that nuclear chromatin structure is better preserved after ethanol fixation than after formalin fixation, since AFA consists of 75 % ethanol (plus 18 % water, 5 % acetic acid, and 2 % formaldehyde solution, meaning 8 g formaldehyde/L). With regard to PAXgene®, our results are concordant to the results from Kap et al. [22], who reported on well-preserved human tissue morphology after PAXgene® fixation in most of the cases.

However, in our study, tissue morphology of xenografts after fixation with HOPE® was not preserved as well as after standard formalin fixation, in contrast to recently published results of fixation of human tissue with HOPE® [17, 18], despite extensive testing of different parameters following manufacturer’s recommendations. The artificial fissures of HOPE®-fixed tissue, like in our study, have also been mentioned from Olert et al. [12].

### Immunohistochemical detectability in alternatively fixed tissue

Immunohistochemical detectability of EGFR, IGF-1R, and phospho-HER2 in our study was the best for tissue fixed with standard formalin throughout all xenograft tumor models. We observed that fixation with AFA (with or without ultrasound sonication) and PAXgene® led to a more intense and quantitative staining of EGFR than formalin fixation, were nearly comparable to formalin fixation for IGF-1R analyses, but surprisingly gave no significant signal in immunohistochemical detection of p-HER2. Our results are consistent to the results of the study on EGFR detectability in alternatively fixed tumor tissue from Atkins et al. [7]. They describe that staining of EGFR in tissue fixed with AFA for 24 h and buffered formalin for 24 h had a comparable performance up to 9 months storage after fixation and that EGFR staining in AFA 24 h-fixed tissue was slightly better compared to buffered formalin-fixed tissue after 12 months storage. However, our additionally performed immunohistochemistry of IGF-1R and p-HER2 in AFA- and formalin-fixed tissue emphasize that not in all cases is AFA as powerful as formalin as a fixative in histopathology.

Especially the unavailable signal for detection of the phospho-site of HER-2 in AFA-fixed tissue does not concur in the experience that total HER2 IHC shows a higher signal in alcohol-fixed specimens. This has to be seen critical in the background of the comparison of results obtained with the non-formalin fixatives to formalin, because there are indications that probing phosphorylated states of therapeutical targets might be more precise in predicting therapeutic response, as presence of phosphorylation surrogates an actively signaling pathway. Therefore, one should carefully validate the performance of alcohol-based fixatives when using it as an alternative to formalin in immunohistochemistry in each case.

For HOPE® fixation we could not achieve IHC results comparable to formalin fixation concerning the three selected receptors. The reasons might lie in the HOPE® fixation procedure for some reason not being amenable to the xenograft model system. Furthermore, we cannot concur with the statement from Olert et al. [12] and Goldman et al. [30] that HOPE®-fixed tissues do not require any pretreatment for immunohistochemistry since we only detected IGF-1R and p-HER2 with IHC in HOPE®-fixed tissue after antigen retrieval with CC1 buffer and CC2 buffer, respectively.

The best results in comparison to standard formalin fixation were achieved with formalin-ultrasound fixation in our study, whereupon staining of p-HER2 in formalin-ultrasound-fixed tissue was not as quantitative as in standard formalin-fixed tissue. However, we do not see underfixation of tissue with formalin-ultrasound as an issue here because detectability of EGFR and IGF-1R was consistently comparable to formalin fixation without ultrasound (standard formalin), but we assume that ultrasound itself could be a reason for the difference. Besides the enhanced reactivity and diffusion of formaldehyde in the tissue [25–27], ultrasound treatment may also trigger chemical reactions or may influence chemical reactions in certain directions [31–34]. One hypothesis could be that phosphorylated proteins are more sensitive to ultrasound treatment than other proteins.

### IHC methodology and alternative fixatives

On the one hand, the often-stated better protein quality and antigenicity of alternatively fixed tissue [4, 5, 9, 11, 35, 36] in our opinion, has to be limited. Our results (improved detection of EGFR with protease treatment in AFA/PAXgene®-fixed tissue, improved detection of IGF-1R with CC1 buffer incubation in AFA/PAXgene®/HOPE®-fixed tissue) imply that also for alternatively fixed tissue, an epitope unmasking pretreatment—the so-called antigen retrieval—can improve immunohistochemical detectability. Similar results have been reported from Nassiri et al. [20] in a study on the alternative fixative UMFIX and from Prento and Lyon [21] in a study on several alternative fixatives. Besides the need for optimized antigen retrieval for alternatively fixed tissue, we observed that one-step on-instrument deparaffinization from alternatively fixed tissue on the BenchMark XT platform led to worse results in immunohistochemistry.

On the other hand, we applied IHC assays, which had been optimized for standard formalin-fixed tissue and thus probably probe denatured epitopes, to alternatively fixed tissue, as we wanted to test alternative fixation methods with regard to performance in routine histopathology. A potential superiority of the alternative fixatives in preserving immuno-detectability of native or conformational epitopes, that might be lost during standard formalin fixation, remains to be elucidated in further studies.

In addition to continuative IHC analyses of more proteins, also comprehensive techniques focusing on detection of various proteins present in fixed tissue, like proteomics-based approaches, could be beneficial [37, 38]. Such approaches have already been applied for the fixative PAXgene® [23] and are showing great potential. Nevertheless, the differing results of alternative fixatives to buffered formalin in IHC could be an indicator, that also with other protein/immuno-based methods (proteomics like MALDI mass spectrometry and protein microarrays or also Western Blot and ELISA) a careful validation and interpretation of the collected data has to be carried out.

## Conclusions

In summary, fixation of xenograft tissue with standard formalin resulted in the best immunohistochemical detectability of the chosen membrane receptors when deparaffinization is performed with the BenchMark XT autostainer. When manual deparaffinization is applied, alternatively fixed tissues show both more intense staining (EGFR) and decreased staining (IGF-1R, p-HER2). While most of the alternative fixatives preserved histomorphology, HOPE®-fixed tissue showed decreased morphology. With regard to clinical routine, it is important to know that IHC staining in alternatively fixed tissue may be dependent on the deparaffinization technique (manual vs. automatic) used. Therefore, the use of alternative fixation methods comes with the need to verify the performance of IHC assays individually for each target.

## Electronic supplementary material

Below is the link to the electronic supplementary material.

Supplementary file 1 Supplementary file 1 shows the detailed methodology of the study (materials and methods) including samples, fixatives, fixation protocols, and procedure of analyses. (PDF 27 kb)

Supplementary file 2 Supplementary file 2 shows the staining protocols used for staining of EGFR, IGF-1R, and p-HER2 on the BenchMark XT autostainer. (PDF 11 kb)

Supplementary file 3 Supplementary file 3 shows details on methodology with representative results of method optimization by means of deparaffinization technique (manual vs. automatic deparaffinization) and antigen retrieval. (PDF 754 kb)

Supplementary file 4 Supplementary file 4 shows complete scoring of immunohistochemistry performed with all differently fixed xenografts. (PDF 119 kb)

## References

[1] Blum F (1893). Der formaldehyd als haertungsmittel. Z wiss Mikrosk.

[2] Blum F (1896). Ueber wesen und wert der formolhärtung. Anat Anz.

[3] Foster CS, Gosden CM, Ke YQ (2006). Primer: tissue fixation and preservation for optimal molecular analysis of urologic tissues. Nat Clin Pract Urol.

[4] Warmington AR, Wilkinson JM, Riley CB (2000). Evaluation of ethanol-based fixatives as a substitute for formalin in diagnostic clinical laboratories. J Histotechnol.

[5] Gillespie JW, Best CJ, Bichsel VE, Cole KA, Greenhut SF, Hewitt SM, Ahram M, Gathright YB, Merino MJ, Strausberg RL, Epstein JI, Hamilton SR, Gannot G, Baibakova GV, Calvert VS, Flaig MJ, Chuaqui RF, Herring JC, Pfeifer J, Petricoin EF, Linehan WM, Duray PH, Bova SG, Emmert-Bucket MR (2002). Evaluation of non-formalin tissue fixation for molecular profiling studies. Am J Pathol.

[6] Srinivasan M, Sedmak D, Jewell S (2002). Effect of fixatives and tissue processing on the content and integrity of nucleic acids. Am J Pathol.

[7] Atkins D, Reiffen KA, Tegtmeier CL, Winther H, Bonato M, Störkel S (2004). Immunohistochemical detection of EGFR in paraffin-embedded tumor tissues: variation in staining intensity due to choice of fixative and storage time of tissue sections. J Histochem Cytochem.

[8] Gugic D, Nassiri M, Nadji M, Morales A, Vincek V (2007). Novel tissue preservative and tissue fixative for comparative pathology and animal research. J Exp Anim Sci.

[9] Burns JA, Li Y, Cheney CA, Ou Y, Franlin-Peifer LL, Kuklin N, Zhang ZQ (2009). Choice of fixative is crucial to successful immunohistochemical detection of phosphoproteins in paraffin-embedded tumor tissues. J Histochem Cytochem.

[10] Ananthanarayanan V, Pins MR, Meyer RE, Gann PH (2005). Immunohistochemical assays in prostatic biopsies processed in Bouin's fixative. J Clin Pathol.

[11] Van der Loos CM (2007). A focus on fixation. J Biotech Histochem.

[12] Olert J, Wiedorn KH, Goldmann T, Kühl H, Mehraein Y, Scherthan H, Niketeghad F, Vollmer E, Müller MA, Müller-Navia J (2001). HOPE fixation: a novel fixing method and paraffin-embedding technique for human soft tissues. Pathol Res Pract.

[13] Goldman T, Wiedorn KH, Kühl H, Olert J, Branscheid D, Pechovsky D, Zissel G, Galle J, Müller-Quernheim J, Vollmer E (2002). Assessment of transcriptional gene activity in situ by application of HOPE-fixed, paraffin embedded tissues. Pathol Res Pract.

[14] Wiedorn KH, Olert J, Stacy RAP, Goldman T, Kühl H, Matthus J, Vollmer E, Bosse A (2002). HOPE—a new fixing technique enables preservation and extraction of high molecular weight DNA and RNA of >20 kb from paraffin embedded tissues. Pathol Res Pract.

[15] Sen Gupta R, Hillemann D, Kubica T, Zissel G, Müller-Quernheim J, Galle J, Vollmer E, Goldmann T (2003). HOPE-Fixation enables improved PCR-based detection and differentiation of *Mycobacterium tuberculosis* complex in paraffin-embedded tissues. Pathol Res Pract.

[16] Goldmann T, Burgemeister R, Sauer U, Loeschke S, Lang D, Branscheid D, Zabel P, Vollmer E (2006). Enhanced molecular analyses by combination of the HOPE-technique and laser microdissection. Diagn Pathol.

[17] Kothmaier H, Rohrer D, Stacher E, Quehenberger F, Becker KF, Popper HH (2011). Comparison of formalin-free tissue fixatives. A proteomic study testing their application for routine pathology and research. Arch Pathol Lab Med.

[18] Braun M, Menon R, Nikolov P, Kirsten R, Petersen K, Schilling D, Schott C, Gundisch S, Fend F, Becker KF, Perner S (2011). The HOPE fixation technique—a promising alternative to common prostate cancer biobanking approaches. BMC Cancer.

[19] Nadji M, Nassiri M, Vincek V, Kanhoush R, Morales AR (2005). Immunohistochemistry of tissue prepared by a molecular-friendly fixation and processing system. Appl Immunohistochem Mol Morphol.

[20] Nassiri M, Ramos S, Zohourian H, Vincek V, Morales AR, Nadji M (2008). Preservation of biomolecules in breast cancer tissue by a formalin-free histology system. J Clin Pathol.

[21] Prento P, Lyon H (1997). Commercial formalin substitutes for histopathology. J Biotech Histochem.

[22] Kap M, Smedts F, Oosterhuis W, Winther R, Christensen N, Reischauer B, Viertler C, Groelz D, Becker KF, Zatloukal K, Langer R, Slotta-Huspenina J, Bodo K, de Jong B, Oelmuller U, Riegman P (2011). Histological assessment of PAXgene tissue fixation and stabilization reagents. PLoS One.

[23] Ergin B, Meding S, Langer R, Kap M, Viertler C, Schott C, Ferch U, Riegman P, Zatloukal K, Walch A, Becker KF (2010). Proteomic analysis of PAXgene-fixed tissues. J Prot Res.

[24] Yasuda K, Yamashita S, Shiozawa M, Aiso S, Yasui Y (1992). Application of ultrasound for tissue fixation: combined use with microwave to enhance the effect of chemical fixation. Acta Histochem Cytochem.

[25] Stephanis CG, Hatiris JG, Mourmouras DE (1998). Acceleration of formaldehyde reactions with proteins due to ultrasound. Ultrason Sonochem.

[26] Chu WS, Furusato B, Wong K, Sesterhenn IA, Mostofi FK, Wei MQ, Zhu Z, Abbondanzo SL, Liang Q (2005). Ultrasound-accelerated formalin fixation of tissue improves morphology, antigen and mRNA preservation. Mod Pathol.

[27] Chu WS, Liang Q, Tang Y, King R, Wong K, Gong M, Wei M, Liu J, Feng SH, Lo SC, Andriko JA, Orr M (2006). Ultrasound-accelerated tissue fixation/processing achieves superior morphology and macromolecule integrity with storage stability. J Histochem Cytochem.

[28] Chen Q, Zou X, Cheng J, Wu J (2007). Application of ultrasound in preparing pathological sections to reduce processing time. Ultrasonics.

[29] von Ahlfen S, Missel A, Bendrat K, Schlumpberger M (2007). Determinants of RNA quality from FFPE samples. PLoS One.

[30] Goldman T, Vollmer E, Gerdes J (2003). What’s cooking? Detection of important biomarkers in HOPE-fixed, paraffin-embedded tissues eliminates the need for antigen retrieval. Corresp Am J Pathol.

[31] Kawai N, Iino M (2003). Molecular damage to membrane proteins induced by ultrasound. Ultrasound Med Biol.

[32] Shkumatov VM, Adzerikho IE, Lesnikovich JA, Cherniavsky EA (2004). Effect of ultrasound on structure and functional properties of antithrombin III and proteins of PPSB complex. Biochemistry (Mosc).

[33] Hickenboth CR, Moore JS, White SR, Sottos NR, Baudry J, Wilson SR (2007). Biasing reaction pathways with mechanical force. Nature.

[34] Filippov AV, Gröbner G, Antzutkin ON (2010). Aggregation of amyloid Aβ(1–40) peptide in perdeuterated 2,2,2-trifluoroethanol caused by ultrasound sonication. Magn Reson Chem.

[35] Bassarova AV, Popov AA (1998). Immunohistochemical detection of p53—effect of fixation and methods of antigen retrieval. Folia Histochem Cytobiol.

[36] Goldstein NS, Hewitt SM, Taylor CR, Yaziji H, Hicks DG (2007). Recommendations for improved standardization of immunohistochemistry. Appl Immunohistochem Mol Morphol.

[37] Becker KF, Taylor CR (2011). “Liquid morphology”: immunochemical analysis of proteins extracted from formalin-fixed paraffin-embedded tissues: combining proteomics with immunohistochemistry. Appl Immunohistochem Mol Morphol.

[38] Rauser S, Deininger SO, Suckau D, Höfler H, Walch A (2010). Approaching MALDI molecular imaging for clinical proteomic research: current state and fields of application. Expert Rev Proteomics.

