# Supplementary material for: Systematic comparison of tissue fixation with alternative fixatives to conventional tissue fixation with buffered formalin in a xenograft-based model
Source: Virchows Arch. 2012 Jul 20;461(3):259–69. doi: 10.1007/s00428-012-1248-5 (PMC3432218; doi:10.1007/s00428-012-1248-5)
Supplement: Supplementary file 1 — shows the detailed methodology of the study (materials and methods) including samples, fixatives, fixation protocols, and procedure of analyses. (PDF 27 kb) [file 428_2012_1248_MOESM1_ESM.pdf]

*Systematic comparison of tissue fixation with alternative fixatives to conventional tissue fixation with buffered formalin in a xenograft based model*. Virchows Archiv. Thorben Nietner, Tiantom Jarutat, Alfred Mertens*
* Roche Diagnostics GmbH, 82377 Penzberg, Germany, alfred.mertens@roche.com

## Supplementary file 1

## Materials and Methods – Details on methodology

### Xenograft Tumor Samples

All samples were obtained from mouse xenograft tissue models of three human cancer cell lines. Xenografts were generated by implanting 5 x 106 COLO-205, OVCAR-5 and NCI-H322M cells suspended in 100 µL of phosphate buffered saline (PBS) subcutaneously in both flanks of SCID-BEIGE mice. After tumor growth to ~400 $mm^3$ (range from 50 to 1100 mg), samples were harvested for fixation and divided into two equally sized pieces with a maximal ischemia time of 60 seconds. One half underwent fixation in standard formalin and the other half in one of the alternative fixatives, as described below. For every alternative fixative at least five xenografts of every cell line from five different mice were generated. Xenograft studies were reviewed and approved by the Government of Upper Bavaria as demanded by the German Animal Welfare Law.

### Fixation Protocols and Fixatives

Fixation of xenograft tumors was optimized for all alternative fixatives in a pre-screen testing of different fixation conditions including fixation time and fixation temperature. Final fixation conditions were selected with regard to optimal tissue morphology obtained by H&E staining of all differently fixed tissues (data not shown). For subsequent experiments (after the pre-screen), tumor samples were fixed for 24 h in 10% v/v buffered formalin (standard formalin; 3.9% w/v formaldehyde; No. 3933.9020, Mallinckrodt Baker; Deventer, Netherlands), for 24 h in Acidified Formal Alcohol (AFA; VWR International; Darmstadt, Germany), for 7 h in 10% v/v buffered formalin with ultrasound exposure (formalin-ultrasound), for 7 h in Acidified Formal Alcohol with ultrasound exposure (AFA-ultrasound) and for a total of 5 h in PAXgene® Tissue Containers, split up in 3 h in Tissue Fix Solution and 2 h in Tissue Stabilizer Solution (PreAnalytiX; Hombrechtikon, Switzerland).

The ultrasound fixation was performed with an USE 33 apparatus (MEDITE; Burgdorf, Germany) according to manufacturer's recommendations at 17.0 °C in 20 mL High Performance Glass Vials (PerkinElmer; Waltham, USA). During fixation, all sample containers were kept in motion to ensure mixing of the fixation solution. After fixation, samples were put in tissue cassettes (Leica; Wetzlar, Germany), excessive fixative was removed by rinsing with water, and samples were processed for 3x 90 min with 70% ethanol, 2x 90 min with 95% ethanol, 2x 90 min with 100% ethanol, 2x 60 min with xylene and 4x 60 min with paraffin (60 °C) in a tissue processor (Sakura; Tokyo, Japan). Dehydration was performed after fixation without any delay. Dehydrated tissues were embedded in Shandon Histoplast paraffin (Thermo Scientific; Cheshire, UK) at 60 °C and stored at 4 °C. Storage temperature of 4 °C was chosen by reason of subsequent RNA analysis (not in this study) as von Ahlfen et al [29] already showed results on degradation of RNA in embedded formalin fixed tissue when storing at room temperature.

The HOPE® fixation (DCS Dr. Christian Sartori; Hamburg, Germany) was performed according to manufacturer's recommendations for 16 h in HOPE® I Solution, 2 h in HOPE® II Solution, dehydration for 3x 2 h in acetone (≥ 99.5% p.a.; Mallinckrodt Baker; Deventer, Netherlands) at 4 °C and paraffinization for 5 h in low melting paraffin at 56 °C (DCS; Hamburg, Germany). The fixed tissues were then embedded in low melting paraffin at 56 °C and stored at 4 °C.

### Morphology

Firstly Xenograft specimens were cut into sections of 3 µm thickness and mounted on Superfrost® plus glass slides (Menzel; Braunschweig, Germany). After drying the slides over night at 37 °C H&E staining was performed with an Tissue-Tek® DRS™ 2000 stainer (Sakura; Tokyo, Japan) using Mayer's hematoxylin and Eosin Y. Also deparaffinization (from xylene over 100%, 90%, 80% ethanol to 70% ethanol [ethanol p.a: 99,8%, denatured with 0.8-1.3% Ethylmethyl-ketone] and dehydration (from water over 70%, 80%, 90 % and 100% ethanol to xylene) were performed with the Tissue-Tek® stainer. After dehydration, slides were coverslipped using EUKITT® (O.Kindler; Freiburg, Germany).

### Immunohistochemistry

Three therapeutically targeted membrane receptors were chosen as IHC markers. For the selected xenograft models, IHC staining of these membrane receptors ranged from low intensity to high intensity when using standard formalin fixation. Therefore, comparability of alternative fixatives with standard formalin fixation was ensured.

The selection complied with established IHC assays of robust antibodies which were all able to perform on a BenchMark XT instrument (Ventana Medical Systems; Tucson, USA) and resulted in staining of EGFR, IGF-1R and p-HER2 with specific antibodies (Ventana Medical Systems; Tucson, USA and Cell Signaling Technology; Danvers, USA. cf. Table 2). Negative controls were performed using IgG control antibodies of the particular species (Table 2).

Xenograft specimens were cut into sections of 4 µm thickness and mounted on Superfrost® plus glass slides (Menzel; Braunschweig, Germany). After drying the slides over night at 37 °C, sections were stained on

a BenchMark XT instrument. Sections were incubated with anti-EGFR antibody for 32 min, with anti-IGF-1R antibody for 16 min and with anti-p-HER2 antibody for 60 min. The antibody-antigen complex was detected by an iView DAB Detection Kit in the case of EGFR and by an UltraView Universal Detection Kit in the case of IGF-1R and p-HER2 using 3,3'-diaminobenzidine (DAB) as the substrate (see Supplementary file 2 for detailed staining protocols). Counterstaining was achieved using Hematoxylin II and Bluing Reagent (all reagents from Ventana Medical Systems). After staining on the BenchMark XT, slides were rinsed with soap water and dehydrated from 70% ethanol over 80%, 90% and 100% ethanol to xylene and then coverslipped using EUKITT® (O.Kindler; Freiburg, Germany).

Because IHC assays with the utilized antibodies were initially established for FFPET, immunohistochemical staining methods were first optimized on xenografts from COLO-205 cells for each alternative fixative by varying assay parameters such as antigen retrieval and deparaffinization. Tissue fixed with standard formalin was analyzed with the standard method established for FFPET on the BenchMark XT instrument with an automatic deparaffinization process and an antigen retrieval of 8min with protease 1 (Ventana Medical Systems) for the detection of EGFR and 60 min with CC1 buffer (Ventana Medical Systems) for the detection of IGF-1R and p-HER2. For HOPE® fixed tissue, we applied standard antigen retrieval with protease 1 and CC1 and as well antigen retrieval with CC2 according to manufacturer's recommendation.

Besides automatic deparaffinization with a proprietary reagent at temperatures around 76 °C (BenchMark XT), manual deparaffinization was performed with xylene to ethanol (90%, 80%, 70%) and phosphate buffered saline (PBS) for all alternatively fixed samples as well. After manual deparaffinization, rehydrated specimens were immediately placed onto the slide pads (thermoflex pads) and covered with PBS to protect sections against over-drying. All sections from HOPE® fixed material were deparaffinized in a separate manner (from 2-propanol to acetone to PBS) according to manufacturer's recommendation.

## IHC Scoring System

All specimens of alternatively fixed tissues were compared with the matched specimens of standard formalin fixed tissue belonging to the same xenograft tumor. The membrane staining was evaluated by an experienced pathologist with respect to both the intensity and the quantity and separately scored from (0) to (3): (0) no staining; (1) weaker overall intensity than standard formalin fixed tissue (intensity) or fewer cells stained than in standard formalin fixed tissue (quantity); (2) equivalent to standard formalin fixed tissue (intensity or quantity); (3) stronger than standard formalin fixed tissue (intensity) or more cells stained than in standard formalin fixed tissue (quantity). The scoring system was adopted similar to the one published by Atkins et al. [7]. Furthermore, other morphological criteria such as counterstaining and preservation of tissue structures were evaluated to detect inappropriate staining methods or inappropriate fixatives. Evaluation of IHC experiments with complete scoring is included in Supplementary file 4.

Table 2 Antibodies used for Immunohistochemistry

| **Antibody** | **Vendor** | **Host species** | **Concentration used** |
|---|---|---|---|
| CONFIRM anti-EGFR (3C6)<br>CONFIRM anti-IGF-1R (G11)<br>anti-Phosphor-HER2/ErbB2 (Tyr1221/1222) | Ventana (Tucson, USA)<br>Ventana (Tucson, USA)<br>Cell Signaling (Danvers, USA) | Mouse<br>Rabbit<br>Rabbit | 1 µg/mL<br>1.7 µg/mL<br>0.15 µg/mL |
| IgG1, isotype Control Mouse<br>IgG (DA1E, #3900), isotype Control Rabbit | R&D (Minneapolis, USA)<br>Cell Signaling (Danvers, USA) | Mouse<br>Rabbit | Equivalent to antibody concentration |
