# Supplementary material for: Systematic comparison of tissue fixation with alternative fixatives to conventional tissue fixation with buffered formalin in a xenograft-based model
Source: Virchows Arch. 2012 Jul 20;461(3):259–69. doi: 10.1007/s00428-012-1248-5 (PMC3432218; doi:10.1007/s00428-012-1248-5)
Supplement: Supplementary file 2 — shows the staining protocols used for staining of EGFR, IGF-1R, and p-HER2 on the BenchMark XT autostainer. (PDF 11 kb) [file 428_2012_1248_MOESM2_ESM.pdf]

Systematic comparison of tissue fixation with alternative fixatives to conventional tissue fixation with buffered formalin in a xenograft based model. Virchows Archiv. Thorben Nietner, Tiantom Jarutat, Alfred Mertens*
* Roche Diagnostics GmbH, 82377 Penzberg, Germany, alfred.mertens@roche.com

Supplementary file 2 - staining protocols

BenchMark-staining protocol - EGFR (3C6) - XT IVIEW DAB v3

Method F_8E (Standard), Protocol Summary

Program-No.	Selected Commands	Notes
1	Paraffin [selected]	
2	Deparaffinization [selected]<br>(4 min 75°C; 4 min 76°C)	For nP-Methods program-No. 2 was not selected (Deparaffinization [not selected])
3	Enzyme	for HOPE®-fixed specimens demasking was performed selecting Conditioner #2
4	Apply One Drop of [PROTEASE 1] (Enzyme), Apply Coverslip, and Incubate for [8 Minutes]	PROTEASE 1 treatment was selected for different incubation times according to particular method
5	Antibody [selected]	
6	Apply One Drop of [EGFR (3C6)] (Antibody), and Incubate for [0 Hr 32 Min]	
7	Counterstain [selected]	
8	Apply One Drop of [HEMATOXYLIN II] (Counterstain), Apply Coverslip, and Incubate for [4 Minutes]	
9	Post Counterstain [selected]	
10	Apply One Drop of [BLUING REAGENT] (Post Counterstain); Apply Coverslip, and Incubate<br>for [4 Minutes]	

BenchMark-staining protocol - IGF-1R (G11) - XT ultraView DAB v3

Method F_60CC1 (Standard), Protocol Summary

Program-No.	Selected Commands	Notes
1	Paraffin [selected]	
2	Deparaffinization [selected]<br>(4 min 75°C; 4 min 76°C)	For nP-Methods program-No. 2 was not selected (Deparaffinization [not selected])
3	Cell Conditioning [selected]	
4	Conditioner #1 [selected]	for HOPE®-fixed specimens demasking was also performed selecting Conditioner #2
5	Mild CC1 [selected]	CC1 treatment was selected for different incubation times according to particular method
6	Standard CC1 [selected]	
7	Ab Incubation Temperatures [selected]	
8	37 C Ab Inc. [selected]	
9	Antibody [selected]	
10	Apply One Drop of [anti-IGF-1R (G11)] (Antibody), and Incubate for [0 Hr 16 Min]	
11	Counterstain [selected]	
12	Apply One Drop of [HEMATOXYLIN II] (Counterstain), Apply Coverslip, and Incubate for [4 Minutes]	
13	Post Counterstain [selected]	
14	Apply One Drop of [BLUING REAGENT] (Post Counterstain); Apply Coverslip, and Incubate<br>for [4 Minutes]	

BenchMark-staining protocol - p-HER2 - XT ultraView DAB v3

Method F_60CC1 (Standard), Protocol Summary

Program-No.	Selected Commands	Notes
1	Paraffin [selected]	
2	Deparaffinization [selected]<br>(4 min 75°C; 4 min 76°C)	For nP-Methods program-No. 2 was not selected (Deparaffinization [not selected])
3	Cell Conditioning [selected]	
4	Conditioner #1 [selected]	for HOPE®-fixed specimens demasking was also performed selecting Conditioner #2
5	Mild CC1 [selected]	CC1 treatment was selected for different incubation times according to particular method
6	Standard CC1 [selected]	
7	Titration [selected]	
8	***** Hand Apply (Primary Antibody), and Incubate for [1 Hour] *****	Incubation of primary antibody at 37°C
9	Counterstain [selected]	
10	Apply One Drop of [HEMATOXYLIN II] (Counterstain), Apply Coverslip, and Incubate for [8 Minutes]	
11	Post Counterstain [selected]	
12	Apply One Drop of [BLUING REAGENT] (Post Counterstain); Apply Coverslip, and Incubate<br>for [8 Minutes]	
