# Supplementary material for: Systematic comparison of tissue fixation with alternative fixatives to conventional tissue fixation with buffered formalin in a xenograft-based model
Source: Virchows Arch. 2012 Jul 20;461(3):259–69. doi: 10.1007/s00428-012-1248-5 (PMC3432218; doi:10.1007/s00428-012-1248-5)
Supplement: Supplementary file 3 — shows details on methodology with representative results of method optimization by means of deparaffinization technique (manual vs. automatic deparaffinization) and antigen retrieval. (PDF 754 kb) [file 428_2012_1248_MOESM3_ESM.pdf]

Systematic comparison of tissue fixation with alternative fixatives to conventional tissue fixation with buffered formalin in a xenograft based model. Virchows Archiv. Thorben Nietner, Tiantom Jarutat, Alfred Mertens*
* Roche Diagnostics GmbH, 82377 Penzberg, Germany, alfred.mertens@roche.com

Supplementary file 3

Details on methodology - deparaffinization and antigen retrieval

Deparaffinization of slides for Immunohistochemistry

In Figure 6 the differences in staining of EGFR after automatic (Figure 6, B+C) and manual (Figure 6, E+F) deparaffinization are repre-sentatively shown for COLO-205 xenografts fixed with AFA for 24h without antigen retrieval (Figure 6, B+E) and with antigen retrieval (Figure 6, C+F).

The influence of deparaffinization on IHC staining of standard formalin fixed tissue was negligible as can be seen in Figure 6 for EGFR staining of COLO-205 xenograft fixed with standard formalin after automatic (Figure 6, A) and manual (Figure 6, D) deparaffinization. In only a few cases, slightly weaker or stronger stainings between manual and automatic deparaffinization were observed for formalin fixed specimens and were supposed to be due to methodical variations (cf. Supplementary file 4).

In the study we first examined COLO-205 xenografts and, only here, we compared automatic vs. manual deparaffinization of standard formalin fixed specimens. Because the difference in IHC scoring for automatically and manually deparaffinized standard formalin fixed specimens was negligible, we continued to compare all IHC slides from alternatively fixed tissue to automatically deparaffinized IHC slides from standard formalin fixed tissue (Figure 7). IHC staining of alternatively fixed xenografts from xenograft models OVCAR-5 and NCI-H322M were performed with optimized staining methods which had been identified for COLO-205 xenografts in a first step (Figure 7).

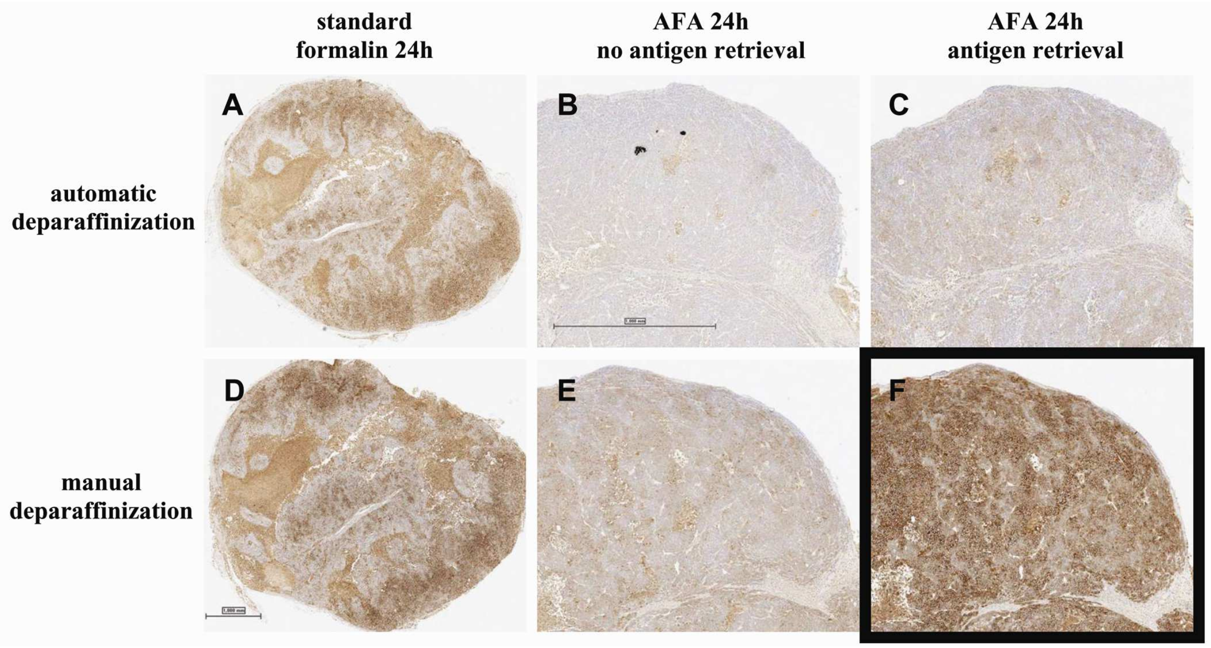

Fig. 6

IHC staining with anti-EGFR of COLO-205 xenograft tissue fixed with standard formalin 24h and AFA 24h. Differences were observed for automatic vs. manual deparaffinization and for application of antigen retrieval. Optimal results for tissue fixed with AFA 24h were achieved with manual deparaffinization and application of antigen retrieval (black frame, picture F). Scale bar shows 1000µm.

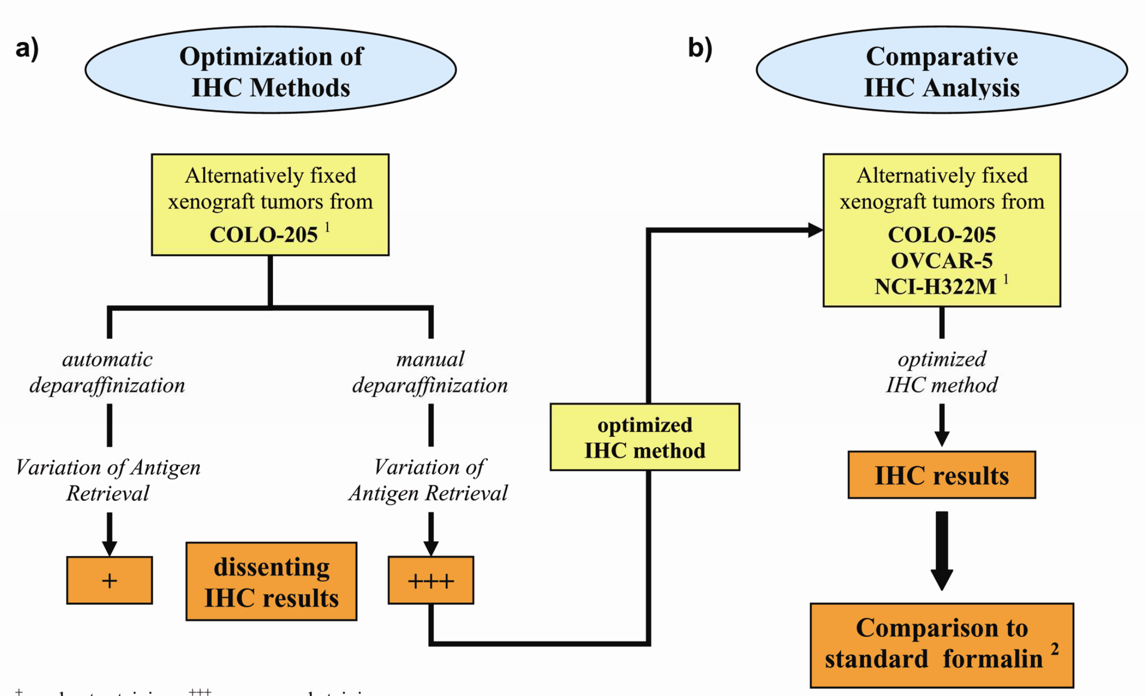

+ moderate staining +++ pronounced staining
[1] PAXgene®, AFA, AFA-ultrasound, formalin-ultrasound; HOPE® was always deparaffinized manually according to manufacturer's recommendations.
[2] standard formalin fixed tissue showed comparable results after manual and automatic deparaffinization and was deparaffinized automatically for comparative IHC analysis. Antigen retrieval was overtaken from established procedures for standard formalin fixed tissue.

Fig. 7
IHC analysis. (a) first step: optimization of IHC staining methods with respect to deparaffinization and antigen retrieval and (b) second step: comparison of alternative fixatives and standard formalin.

Antigen retrieval

In addition to the optimization of the deparaffinization process, we selected antigen retrieval leading to optimized IHC results (more intense staining) for the detection of every membrane receptor (EGFR, IGF-1R and p-HER2) in every alternatively fixed tissue (Figure 7). Besides staining intensity, IHC results were evaluated with regard to nuclear morphology, counterstaining and tissue preservation.

In most of the cases, antigen retrieval times were shorter or of the same duration as for the use of standard formalin fixed tissue (Table 3). However, antigen retrieval improved staining of alternatively fixed tissue as can be seen for example in EGFR staining of a COLO-205 xenograft fixed with AFA for 24h with antigen retrieval (Figure 6, C+F) and without antigen retrieval (Figure 6, B+E).

Table 3 Optimization of IHC staining methods

Fixation	Methods EGFR	Methods IGF-1R	Methods p-HER2
PAXgene®	nP_4 min protease 1	nP_60 min CC1	nP_60 min CC1
AFA	nP_4 min protease 1	nP_60 min CC1	nP_60 min CC1
AFA-ultrasound	nP_4 min protease 1	nP_60 min CC1	nP_60 min CC1
formalin-ultrasound	nP_4 min protease 1	nP_90 min CC1	60 min CC1
HOPE®	not possible	nP_30 min CC1	nP_36 min CC2
standard formalin	8 min protease 1	60 min CC1	60 min CC1

* nP: manual deparaffinization before staining with BenchMark XT
CC1 and CC2: retrieval buffer
