# Supplementary material for: Systematic comparison of tissue fixation with alternative fixatives to conventional tissue fixation with buffered formalin in a xenograft-based model
Source: Virchows Arch. 2012 Jul 20;461(3):259–69. doi: 10.1007/s00428-012-1248-5 (PMC3432218; doi:10.1007/s00428-012-1248-5)
Supplement: Supplementary file 4 — shows complete scoring of immunohistochemistry performed with all differently fixed xenografts. (PDF 119 kb) [file 428_2012_1248_MOESM4_ESM.pdf]

Systematic comparison of tissue fixation with alternative fixatives to conventional tissue fixation with buffered formalin in a xenograft based model.
Virchows Archiv. Thorben Nietner, Tiantom Jarutat, Alfred Mertens*
* Roche Diagnostics GmbH, 82377 Penzberg, Germany, alfred.mertens@roche.com

Supplementary file 4 - IHC scoring

A) Fixation PAXgene® 3h – cell line COLO-205

receptor	IHC-method	tumor 1		tumor 2		tumor 3		tumor 4		tumor 5		comments
		I	Q	I	Q	I	Q	I	Q	I	Q	
EGFR	PAX_0E	0	0	0	0	0	0	0	0	0	0	
	PAX_4E	1	1_{focal}	1	1_{focal}	1	1_{focal}	1	1_{focal}	0	0	
	PAX_8E	1	1_{focal}	1	1_{focal}	1	1_{focal}	1	1_{focal}	0	0	faded nuclei, getting pale blue
	PAX_nP_0E	1	1	1	1	1	1	2	1	1	1_{focal}	
	PAX_nP_4E	2	2-3	2	2-3	2	2	3	2	3*	3*	
	PAX_nP_8E	2	2	2	2	2	2	3	2	3*	3*	decreased morphology, nuclei not apparent/not blue
	F_8E (BenchMark)	2	2 (65%)	2	2 (70%)	2	2 (60%)	2	2 (60%)	2	2 (30%)	distinct difference between BenchMark and manual deparaffinization was only detectable for tumor 5
	F_nP_8E (manual)	2	2 (65%)	2	1 (60%)	2	2 (60%)	2	2 (60%)	3	3 (80%)	
	* in comparison to F_nP_8E (manual) scoring is I = 2; Q = 2											
IGF-1R	PAX_0CC1	0	0	0	0	0	0	0	0	0	0	
	PAX_8CC1	1	1_{focal}	1	1_{focal}	1	1_{focal}	1	1	1	1_{focal}	
	PAX_30CC1	1	1	1	1	1	1	1	1	nicht auswertbar		
	PAX_60CC1	1	1	1	1	1	1	1	1	1-2	1	
	PAX_nP_0CC1	0	0	0	0	0	0	0	0	0	0	
	PAX_nP_8CC1	1	1	1	1	1	1	1	1	1	1	
	PAX_nP_30CC1	2	1	1	2	1	1-2	2	2	2	1-2	

A) fixation PAXgene® 3h - cell line COLO-205 (continuation)

receptor	IHC-method	tumor 1 I	tumor 1 Q	tumor 2 I	tumor 2 Q	tumor 3 I	tumor 3 Q	tumor 4 I	tumor 4 Q	tumor 5 I	tumor 5 Q	comments
IGF-1R	PAX_nP_60CC1	2	2	2	1-2	2	1-2	1-2	2	2	2	
	PAX_nP_90CC1	2	2	2	1-2	1-2	1	1-2	2	2	2	
	F_60CC1 (BenchMark)	2	2	2	2	2	2	2	2	2	2	medium - strong staining in all tumors 100% of cells stained
	F_nP_60CC1 (manual)	2	2	2	2	2	2	2	2	2-3	2	
p-HER2	PAX_0CC1	0	0	0	0	0	0	0	0	0	0	
	PAX_8CC1	0	0	0	0	0	0	0	0	0	0	
	PAX_30CC1	0	0	0	0	0	0	0	0	0	0	
	PAX_60CC1	0	0	0	0	0	0	0	0	0	0	
	PAX_nP_0CC1	0	0	0	0	0	0	0	0	0	0	
	PAX_nP_8CC1	0	0	1	1	0	0	1	1	0	0	background stainings in few cases, which were however not looking like isotype controls
	PAX_nP_30CC1	0	0	1	1	1	1	1	1	0	0	
	PAX_nP_60CC1	0	0	1	1	1	1	1	1	0	0	
	PAX_nP_90CC1	0	0	1	1	1	1	1	1	0	0	
	F_60CC1 (BenchMark)	2	2 (60%)	2	2 (90%)	2	2 (90%)	2	2 (90%)	2 (60%)	2	medium - strong staining
	F_nP_60CC1 (manual)	2	2 (60%)	2	1 (80%)	2	2 (90%)	2	2 (90%)	2 (60%)	2	
	Isotype controls of standard formalin tissue were also weakly stained (unspecific staining)											

scoring: intensity: 0 = no staining; 1 = weaker than standard formalin; 2 = equivalent to standard formalin (BenchMark deparaffinization); 3 = stronger than standard formalin
quantity: 0 = no cells stained (0% of cells); 1focal = less than 10% of cells stained; 2 = equivalent to standard formalin (BenchMark deparaffinization); 3 = more cells stained than in standard formalin; F = standard formalin fixed and paraffin embedded tissue; PAX = tissue fixed with PAXgene® and paraffin embedded; staining quantity for standard formalin is expressed in %
numbers in title of IHC method = time of antigen retrieval expressed in minutes, nP = manual deparaffinization, CC1 = antigen retrieval buffer (Ventana Medical Systems)

A) fixation PAXgene® 3h - cell line OVCAR-5

receptor	IHC-method	tumor 1		tumor 2		tumor 3		tumor 4		tumor 5		comments
		I	Q	I	Q	I	Q	I	Q	I	Q	
EGFR	PAX_nP_4E	3	3	2-3	3	2	3	3	3	3	3	
	F_8E (BenchMark)	2	2* (90 %)	2	2 (95 %)	2	2 (90 %)	2	2 (95 %)	2	2 (95 %)	medium - strong staining
IGF-1R	PAX_nP_60CC1	1	1_{focal}	2	1	2	1	1	1	1-2	1	
	F_60CC1 (BenchMark)	2	2 (40 %)	2	2 (70 %)	2	2 (70 %)	2	2 (60 %)	2	2 (70 %)	medium - strong staining
p-HER2	PAX_nP_60CC1	0	0	0	0	0	0	0	0	0	0	
	F_60CC1 (BenchMark)	2	2 (90 %)	2†	2 (80 %)	2	2 (80 %)	2	2 (80 %)	2	2 (80 %)	medium - strong staining;<br>† weak - medium staining

* very strong unspecific staining of isotype control; evaluation of result limited; isotype controls: weak unspecific staining of mouse tissue (p-HER2)

A) fixation PAXgene® 3h - cell line NCI-H322M

receptor	IHC-method	tumor 1		tumor 2		tumor 3		tumor 4		tumor 5		comments
		I	Q	I	Q	I	Q	I	Q	I	Q	
EGFR	PAX_nP_4E	2	2-3	2	3	2-3	3	2-3	3	2	2	
	F_8E (BenchMark)	2	2 (80 %)	2	2 (80 %)	2	2 (70 %)	2	2 (90 %)	2	2 (80 %)	medium - strong staining
IGF-1R	PAX_nP_60CC1	2	1	2	1	2	1	1-2	1	1-2	1	
	F_60CC1 (BenchMark)	2	2 (95 %)	2	2 (90 %)	2	2 (95 %)	2	2 (90 %)	2	2 (95 %)	medium - strong staining
p-HER2	PAX_nP_60CC1	0	0	0	0	0	0	1	1 focal	0	0	
	F_60CC1 (BenchMark)	2	2 (70 %)	2	2 (80 %)	2	2 (60 %)	2	2 (80 %)	2	2 (90 %)	medium - strong staining

scoring: intensity: 0 = no staining; 1 = weaker than standard formalin; 2 = equivalent to standard formalin (BenchMark deparaffinization); 3 = stronger than standard formalin
quantity: 0 = no cells stained (0% of cells); 1focal = less than 10% of cells stained; 2 = equivalent to standard formalin (BenchMark deparaffinization); 3 = more cells stained than in standard formalin; F = standard formalin fixed and paraffin embedded tissue; PAX = tissue fixed with PAXgene® and paraffin embedded; staining quantity for standard formalin is expressed in %
numbers in title of IHC method = time of antigen retrieval expressed in minutes, nP = manual deparaffinization, CC1 = antigen retrieval buffer (Ventana Medical Systems)

B) fixation AFA 24h - cell line COLO-205

receptor	IHC-method	tumor 1		tumor 2		tumor 3		tumor 4		tumor 5		comments
		I	Q	I	Q	I	Q	I	Q	I	Q	
EGFR	AFA_0E	1	1_{focal}	1	1_{focal}	1	1_{focal}	0	0	0	0	
	AFA_4E	2	1-2	1	1	1	1	1	1	1	1	
	AFA_8E	1	1	1	1	1	1	1*	1*	1*	1*	* nuclei poorly preserved, decreased morphology
	AFA_nP_0E	1-2	1	1	1	1-2	1	2	1	1-2	1	
	AFA_nP_4E	2	3	2-3	3	2	2-3	2	3	3	3	
	AFA_nP_8E	2	2	2-3†	3†	3†	3†	2†	3†	3	3	† destroyed nuclei
	F_8E (BenchMark)	2	2 (45%)	2	2 (35%)	2	2 (50%)	2	2 (30%)	2	2 (30%)	
	F_nP_8E (manual)	1-2	1-2 (40%)	2	2 (35%)	2	2 (50%)	2	2-3 (40%)	2	2-3 (40%)	
IGF-1R	AFA_0CC1	0	0	0	0	0	0	0	0	0	0	
	AFA_8CC1	1	1_{focal}	1	1_{focal}	1	1_{focal}	1	1_{focal}	1	1_{focal}	
	AFA_30CC1	1	1	1	1	1-2	1	1	1	1	1	
	AFA_60CC1	1	1	1-2	1	2	1	1-2	1	1-2	1	
	AFA_nP_0CC1	1	1_{focal}	0	0	0		0	0	0	0	
	AFA_nP_8CC1	1	1_{focal}	1	1_{focal}	1	1_{focal}	1	1_{focal}	1	1	
	AFA_nP_30CC1	2	1	1-2	1	1-2	1	1-2	1	1-2	1	
	AFA_nP_60CC1	1-2	1	2	1	2	1	2	1	2	1	

B) fixation AFA 24h - cell line COLO-205 (continuation)

receptor	IHC-method	tumor 1		tumor 2		tumor 3		tumor 4		tumor 5		comments
		I	Q	I	Q	I	Q	I	Q	I	Q	
IGF-1R	AFA_nP_90CC1	2	1	2	1-2	2	1	2	1	2	1	
	F_60CC1 (BenchMark)	2	2	2	2	2	2	2	2	2	2	medium - strong staining in all tumors 100% of cells stained
	F_nP_60CC1 (manual)	2	2	2	2	2	2	2	2	2	2	
p-HER2	AFA_0CC1	0	0	0	0	0	0	0	0	0	0	
	AFA_8CC1	0	0	0	0	0	0	0	0	0	0	
	AFA_30CC1	0	0	0	0	0	0	0	0	0	0	
	AFA_60CC1	0	0	0	0	0	0	0	0	0	0	
	AFA_nP_0CC1	0	0	1	1	1	1	0	0	1	1	
	AFA_nP_8CC1	0	0	1	1	1	1	0	0	1	1	background stainings partially detectable, however looking like isotype controls
	AFA_nP_30CC1	0	0	1	1	1	1	0	0	1	1	
	AFA_nP_60CC1	0	0	1	1	1	1	0	0	1	1	
	AFA_nP_90CC1	0	0	1	1	1	1	0	0	1	1	
	F_60CC1 (BenchMark)	2	2 (70%)	2	2 (80%)	2	2 (75%)	2	2 (90%)	2	2 (90%)	medium - strong staining
	F_nP_60CC1 (manual)	2	2 (70%)	2	2-3 (90%)	2	2 (75%)	2	1-2 (80%)	3	2 (90%)	
	Isotype controls of standard formalin tissue were also weakly stained (unspecific staining)											

scoring: intensity: 0 = no staining; 1 = weaker than standard formalin; 2 = equivalent to standard formalin (BenchMark deparaffinization); 3 = stronger than standard formalin
quantity: 0 = no cells stained (0% of cells); 1focal = less than 10% of cells stained; 2 = equivalent to standard formalin (BenchMark deparaffinization); 3 = more cells stained than in standard formalin; F = standard formalin fixed and paraffin embedded tissue; AFA = tissue fixed with AFA and paraffin embedded; staining quantity for standard formalin is expressed in %
numbers in title of IHC method = time of antigen retrieval expressed in minutes, nP = manual deparaffinization, CC1 = antigen retrieval buffer (Ventana Medical Systems)

B) fixation AFA 24h - cell line OVCAR-5

receptor	IHC-method	tumor 1		tumor 2		tumor 3		tumor 4		tumor 5		comments
		I	Q	I	Q	I	Q	I	Q	I	Q	
EGFR	AFA_nP_4E	3	2-3 (95 %)	3	3	3	3 (100 %)	3	3	2-3	3	
	F_8E (BenchMark)	2	2 (90 %)	2	2 (80 %)	2	2 (70 %)	2	2 (90 %)	2	2 (95 %)	weak - medium staining; partially heterogeneous
IGF-1R	AFA_nP_60CC1	1-2	1	1-2	1	1-2	1	1-2	1	1	1_{focal}	
	F_60CC1 (BenchMark)	2	2	2	2	2	2	2	2	2	2	medium - strong staining
p-HER2	AFA_nP_60CC1	0	0	0	0	0	0	0	0	0	0	
	F_60CC1 (BenchMark)	2	2 (80 %)	2	2 (90 %)	2	2 (80 %)	2	2 (90 %)	2	2 (90 %)	medium - strong staining; isotyp controls showing weak background staining

B) fixation AFA 24h - cell line NCI-H322M

receptor	IHC-method	tumor 1		tumor 2		tumor 3		tumor 4		tumor 5		comments
		I	Q	I	Q	I	Q	I	Q	I	Q	
EGFR	AFA_nP_4E	3	3	2	2	2	2	3	3	3	3	
	F_8E (BenchMark)	2	2 (70 %)	2	2 (90 %)	2	2 (90 %)	2	2 (60 %)	2	2 (60 %)	(medium-) strong staining
IGF-1R	AFA_nP_60CC1	2	1	1-2	1-2	2	1	1	1	2	1	
	F_60CC1 (BenchMark)	2	2 (90 %)	2	2 (80 %)	2	2 (95 %)	2	2 (90 %)	2	2 (90 %)	weak - medium staining
p-HER2	AFA_nP_60CC1	0	0	0	0	0	0	0	0	0	0	
	F_60CC1 (BenchMark)	2	2 (70 %)	2	2 (70 %)	2	2 (70 %)	2	2 (80 %)	2	2 (90 %)	medium - strong staining

scoring: intensity: 0 = no staining; 1 = weaker than standard formalin; 2 = equivalent to standard formalin (BenchMark deparaffinization); 3 = stronger than standard formalin
quantity: 0 = no cells stained (0% of cells); 1focal = less than 10% of cells stained; 2 = equivalent to standard formalin (BenchMark deparaffinization); 3 = more cells stained than in standard formalin; F = standard formalin fixed and paraffin embedded tissue; AFA = tissue fixed with AFA and paraffin embedded; staining quantity for standard formalin is expressed in %
numbers in title of IHC method = time of antigen retrieval expressed in minutes, nP = manual deparaffinization, CC1 = antigen retrieval buffer (Ventana Medical Systems)

C) fixation AFA/USM 7h - cell line COLO-205

receptor	IHC-method	tumor 1		tumor 2		tumor 3		tumor 4		tumor 5		comments
		I	Q	I	Q	I	Q	I	Q	I	Q	
EGFR	AFA/USM_0E	0	0	1	1 focal	1	1 focal	0	0	0	0	
	AFA/USM_4E	1	1 focal	1	1	1	1	1	1	1	1	
	AFA/USM_8E	1	1	1	1	1	1	1	1	1	1	nuclei poorly preserved, decreased morphology
	AFA/USM_nP_0E	1	1	1-2	1-2	1-2	1	1-2	1	2	1	
	AFA/USM_nP_4E	2	2	3	3	2-3	3	3	3	2-3	3	
	AFA/USM_nP_8E	2	2	2-3	2-3	2	3	3	3	2	3	destroyed nuclei, membrane appears unclear
	F_8E (BenchMark)	2	2 (70%)	2	2 (60%)	2	2 (75%)	2	2 (50%)	2	2 (50%)	isotyp controls showed background staining of mouse tissue
	F_nP_8E (manual)	1	1	2	2	2	1 (50%)	1	1 (20%)	1-2	1 (30%)	
IGF-1R	AFA/USM_0CC1	0	0	0	0	0	0	0	0	0	0	
	AFA/USM_8CC1	1	1 focal	1	1	1	1	1	1	1	1	
	AFA/USM_30CC1	1	1	1-2	1-2	1	1	1	1	1	1	
	AFA/USM_60CC1	1	1	2	1-2	1-2	1	1-2	1	1-2	1	
	AFA/USM_90CC1	1	1	1	1-2	1	1	1	1	1-2	1-2	
	AFA/USM_nP_0CC1	0	0	0	0	1	1 focal	0	0	0	0	
	AFA/USM_nP_8CC1	1	1	1	1	1	1	1	1	1	1	
	AFA/USM_nP_30CC1	1	1	2	1-2	1	1	1-2	1	1	1	

C) fixation AFA/USM 7h - cell line COLO-205 (continuation)

receptor	IHC-method	tumor 1 I	tumor 1 Q	tumor 2 I	tumor 2 Q	tumor 3 I	tumor 3 Q	tumor 4 I	tumor 4 Q	tumor 5 I	tumor 5 Q	comments
IGF-1R	AFA/USM_nP_60CC1	1-2	2	2	2	1-2	1-2	1-2	1-2	1	1-2	
	AFA/USM_nP_90CC1	1-2	2	2	2	1-2	1-2	1-2	1-2	1	1-2	
	F_60CC1 (BenchMark)	2	2	2	2	2	2	2	2	2	2	medium - strong staining in all tumors 100% of cells stained
	F_nP_60CC1 (manual)	2	2	2	2	1-2	2	2	2	2	2	
p-HER2	AFA/USM_0CC1	0	0	0	0	0	0	0	0	0	0	
	AFA/USM_8CC1	0	0	0	0	0	0	0	0	0	0	
	AFA/USM_30CC1	0	0	0	0	0	0	0	0	0	0	
	AFA/USM_60CC1	0	0	0	0	0	0	0	0	0	0	
	AFA/USM_nP_0CC1	0	0	0	0	0	0	0	0	0	0	
	AFA/USM_nP_8CC1	0	0	0	0	0	0	0	0	0	0	weak unspecific background staining
	AFA/USM_nP_30CC1	0	0	0	0	0	0	0	0	0	0	
	AFA/USM_nP_60CC1	0	0	0	0	0	0	0	0	0	0	
	AFA/USM_nP_90CC1	0	0	0	0	0	0	0	0	0	0	
	F_60CC1 (BenchMark)	2	2 (80%)	2	2 (80%)	2	2 (95%)	2	2 (90%)	2	2 (95%)	medium - strong staining
	F_nP_60CC1 (manual)	2	2	1-2	1 (60%)	2	1 (80%)	2	2	2	2	
	Isotype controls of standard formalin tissue were also weakly stained (unspecific staining)											

scoring: intensity: 0 = no staining; 1 = weaker than standard formalin; 2 = equivalent to standard formalin (BenchMark deparaffinization); 3 = stronger than standard formalin
quantity: 0 = no cells stained (0% of cells); 1focal = less than 10% of cells stained; 2 = equivalent to standard formalin (BenchMark deparaffinization); 3 = more cells stained than in standard formalin; F = standard formalin fixed and paraffin embedded tissue; AFA/USM = tissue fixed with AFA-ultrasound 7h and paraffin embedded; staining quantity for standard formalin expressed in %
numbers in title of IHC method = time of antigen retrieval expressed in minutes, nP = manual deparaffinization, CC1 = antigen retrieval buffer (Ventana Medical Systems)

C) fixation AFA/USM 7h - cell line OVCAR-5

receptor	IHC-method	tumor 1		tumor 2		tumor 3		tumor 4		tumor 5		comments
		I	Q	I	Q	I	Q	I	Q	I	Q	
EGFR	AFA/USM_nP_4E	3	3	3	3	2-3	3	2	2	3	3	
	F_8E (BenchMark)	2	2* (95 %)	2	2* (80 %)	2	2 (95 %)	2	2 (100 %)	2	2 (95 %)	strong staining; * heterogeneous staining
IGF-1R	AFA/USM_nP_60CC1	1	1_{focal}	1	1	1	1_{focal}	1	1	1	1	
	F_60CC1 (BenchMark)	2	2 (50 %)	2	2 (80 %)	2	2 (60 %)	2	2 (80 %)	2	2 (70 %)	weak - medium staining
p-HER2	AFA/USM_nP_60CC1	0	0	0	0	0	0	0	0	0	0	
	F_60CC1 (BenchMark)	2	2	2	2	2	2	2	2	2	2	medium staining

C) fixation AFA/USM 7h - cell line NCI-H322M

receptor	IHC-method	tumor 1		tumor 2		tumor 3		tumor 4		tumor 5		comments
		I	Q	I	Q	I	Q	I	Q	I	Q	
EGFR	AFA/USM_nP_4E	3	3	3	3	3	3	3	3	2	1	
	F_8E (BenchMark)	2	2 (60 %)	2	2 (60 %)	2	2 (50 %)	2	2 (60 %)	2	2 (90 %)	medium staining
IGF-1R	AFA/USM_nP_60CC1	1-2	1	1	1	1-2	1	2	1	1	1	
	F_60CC1 (BenchMark)	2	2 (90 %)	2	2 (90 %)	2	2 (90 %)	2	2 (90 %)	2	2 (80 %)	medium - strong staining
p-HER2	AFA/USM_nP_60CC1	0	0	0	0	0	0	0	0	0	0	
	F_60CC1 (BenchMark)	2	2 (70 %)	2	2 (70 %)	2	2 (80 %)	2	2 (90 %)	2	2 (40 %)	medium - strong staining

scoring: intensity: 0 = no staining; 1 = weaker than standard formalin; 2 = equivalent to standard formalin (BenchMark deparaffinization); 3 = stronger than standard formalin
quantity: 0 = no cells stained (0% of cells); 1focal = less than 10% of cells stained; 2 = equivalent to standard formalin (BenchMark deparaffinization); 3 = more cells stained than in standard formalin; F = standard formalin fixed and paraffin embedded tissue; AFA/USM = tissue fixed with AFA-ultrasound 7h and paraffin embedded; staining quantity for standard formalin expressed in %
numbers in title of IHC method = time of antigen retrieval expressed in minutes, nP = manual deparaffinization, CC1 = antigen retrieval buffer (Ventana Medical Systems)

D) fixation F/USM 7h - cell line COLO-205

receptor	IHC-method	tumor 1		tumor 2		tumor 3		tumor 4		tumor 5		comments
		I	Q	I	Q	I	Q	I	Q	I	Q	
EGFR	F/USM_0E	1	1_{focal}	0	0	0	0					
	F/USM_4E	1	1-2	2	1-2	2	2					
	F/USM_8E	2	1-2	2	2	2	2-3					
	F/USM_nP_0E	1	1	0	0	0	0	not evaluable, strong stainings also for isotype controls (leaky mice)				
	F/USM_nP_4E	2	2-3	2	2-3	2	2-3					
	F/USM_nP_8E	2	2	2	2	1	1_{focal}					
	F_8E (BenchMark)	2	2 (70%)	2	2 (60%)	2	2 (30%)					
	F_nP_8E (manual)	2	2	2	1 (40%)	2	2					
IGF-1R	F/USM_0CC1	0	0	0	0	1	1_{focal}					
	F/USM_8CC1	1	1	1	1	1	1					
	F/USM_30CC1	1	1-2	2	1-2	2	1-2					
	F/USM_60CC1	1	1-2	2	2	1-2	1	not evaluable, strong stainings also for isotype controls (leaky mice)				
	F/USM_90CC1	2	2	2	2	2	1-2					
	F/USM_nP_0CC1	0	0	0	0	0	0					
	F/USM_nP_8CC1	1	1	1	1	1	1					
	F/USM_nP_30CC1	2	1-2	2	1-2	1-2	1					

D) fixation F/USM 7h - cell line COLO-205 (continuation)

receptor	IHC-method	tumor 1 I	tumor 1 Q	tumor 2 I	tumor 2 Q	tumor 3 I	tumor 3 Q	tumor 4 I	tumor 4 Q	tumor 5 I	tumor 5 Q	comments
	F/USM_nP_60CC1	2	2	2	2	2	1-2	not evaluable, strong stainings also for isotype controls (leaky mice)				
	F/USM_nP_90CC1	2	2	2	2	2	2					
	F_60CC1 (BenchMark)	2	2	2	2	2	2					medium - strong staining in all tumors 100% of cells stained
	F_nP_60CC1 (manual)	2	2	2	2	2	2					
p-HER2	F/USM_0CC1	0	0	0	0	0	0	not evaluable, strong stainings also for isotype controls (leaky mice)				
	F/USM_8CC1	1	1	1	1	1	1					
	F/USM_30CC1	1-2	1	2	1	2	1-2					
	F/USM_60CC1	2	1	2	1	2	1-2					
	F/USM_nP_0CC1	0	0	0	0	0	0					
	F/USM_nP_8CC1	1	1	1	1	1	1					
	F/USM_nP_30CC1	2	1	2	1	2	1-2					
	F/USM_nP_60CC1	2	1	2	1	1-2	1-2					
	F/USM_nP_90CC1	2	1	2	1	2	1-2					
	F_60CC1 (BenchMark)	2	2 (85%)	2	2 (95%)	2	2 (75%)					
	F_nP_60CC1 (manual)	2	1 (65%)	2	1 (85%)	2	2-3 (90%)					
	Isotype controls of standard formalin tissue were also weakly stained (unspecific staining), but not the tisotype controls of F/USM											

scoring: intensity (I): 0 = no staining; 1 = weaker than standard formalin; 2 = equivalent to standard formalin (BenchMark deparaffinization); 3 = stronger than standard formalin
quantity (Q): 0 = no cells stained (0% of cells); 1focal = less than 10% of cells stained; 2 = equivalent to standard formalin (BenchMark deparaffinization); 3 = more cells stained than in standard formalin; F = standard formalin fixed paraffin embedded tissue; F/USM = tissue fixed with formalin-ultrasound 7h and paraffin embedded; staining quantity for standard formalin expressed in %
numbers in title of IHC method = time of antigen retrieval expressed in minutes, nP = manual deparaffinization, CC1 = antigen retrieval buffer (Ventana Medical Systems)

D) fixation F/USM 7h - cell line OVCAR-5

receptor	IHC-method	tumor 1		tumor 2		tumor 3		tumor 4		tumor 5		comments
		I	Q	I	Q	I	Q	I	Q	I	Q	
EGFR	F/USM_nP_4E	2-3	2	2	2	2	2	2	2	3	3 (95 %)	
	F_8E (BenchMark)	2	2 (100 %)	2	2 (100 %)	2	2 (100 %)	2	2 (100 %)	2	2 (90 %)	strong staining
IGF-1R	F/USM_nP_90CC1	2	1	2	1-2	1-2	1	2	1	2	2	
	F_60CC1 (BenchMark)	2	2* (60 %)	2	2 (50 %)	2	2 (60 %)	2	2 (80 %)	2	2 (70 %)	medium staining; * heterogeneous staining
p-HER2	F/USM_60CC1	2	1	1-2	1	2	1	2	1	2	1	
	F_60CC1 (BenchMark)	2	2	2	2	2	2	2	2	2	2	medium staining

D) fixation F/USM 7h - cell line NCI-H322M

receptor	IHC-method	tumor 1		tumor 2		tumor 3*		tumor 4		tumor 5		comments
		I	Q	I	Q	I	Q	I	Q	I	Q	
EGFR	F/USM_nP_4E	2	2	3	2-3	2	2-3	3	2	3	3	
	F_8E (BenchMark)	2	2 (90 %)	2	2 (90 %)	2	2 (80 %)	2	2 (90 %)	2	2 (80 %)	strong staining
IGF-1R	F/USM_nP_90CC1	2-3	2	2	2	2	2	2	1-2	2	1-2	
	F_60CC1 (BenchMark)	2	2 (70 %)	2	2 (90 %)	2	2 (95 %)	2	2 (90 %)	2	2 (80 %)	medium staining
p-HER2	F/USM_60CC1	2	2	1	1	2	1	2	1	2	1	
	F_60CC1 (BenchMark)	2	2 (60 %)	2	2 (80 %)	2	2 (70 %)	2	2 (70 %)	2	2 (80 %)	medium staining

* result doubtful because also isotype controls showed staining

scoring: intensity (I): 0 = no staining; 1 = weaker than standard formalin; 2 = equivalent to standard formalin (BenchMark deparaffinization); 3 = stronger than standard formalin
quantity (Q): 0 = no cells stained (0% of cells); 1focal = less than 10% of cells stained; 2 = equivalent to standard formalin (BenchMark deparaffinization); 3 = more cells stained than in standard formalin; F = standard formalin fixed and paraffin embedded tissue; F/USM = tissue fixed with formalin-ultrasound 7h and paraffin embedded; staining quantity for standard formalin expressed in %
numbers in title of IHC method = time of antigen retrieval expressed in minutes, nP = manual deparaffinization, CC1 = antigen retrieval buffer (Ventana Medical Systems)

E) fixation HOPE® - cell line COLO-205

receptor	IHC-method	tumor 1		tumor 2		tumor 3		tumor 4		tumor 5		comments
		I	Q	I	Q	I	Q	I	Q	I	Q	
EGFR	HOPE_nP_0CC2	0	0	0	0	0	0	0	0	0	0	
	HOPE_nP_8CC2	1-2	3*	1	2*	1	3*	1	1*	1	1*	
	HOPE_nP_36CC2	1-2	2-3	1-2	3*	1	3*	1	1*	1	1-2*	
	HOPE_nP_60CC2	1-2	1-2*	1	1-2*	1	2*	1	1*	1	1-2*	decreased and partially destroyed morphology
	HOPE_nP_84CC2	1-2	1-2*	1-2	3	1	1-2	1	1	1	1	
	isotype control	1-2	1-2*	1-2	1	1	2*	1	1*	1	1-2*	isotype control consistently stained, results therefore doubtful
	F_8E (BenchMark)	2	2 (30 %)	2	2 (20 %)	2	2 (40 %)	2	2 (30 %)	2	2 (50 %)	isotype control showed unspecific staining of mouse tissue
	F_nP_8E (manual)	not performed										
	* cytoplasmatic staining beside only very weak partial membrane staining											
IGF-1R	HOPE_nP_0CC1	1	1-2	1	1	1	1 focal	1	1 focal	1	1 focal	
	HOPE_nP_8CC1	2-3	1-2	2-3	1	2-3	1	1-2	1	2	1	
	HOPE_nP_30CC1	2-3	1-2	2-3	1-2	3	1-2	2-3	1	2-3	1-2	
	HOPE_nP_60CC1	2-3	1-2	2-3	1-2	3	2	2-3	1	2	1-2	morphology worse than for HOPE_nP_30CC1
	HOPE_nP_90CC1	3	1-2	2-3	1-2	3	1-2	2-3	1	2	1-2	
	F_60CC1 (BenchMark)	2	2	2	2*	2	2	2	2	2	2	medium - strong staining 100% stained; * 95% stained
	F_nP_60CC1 (manual)	not performed										

E) fixation HOPE® - cell line COLO-205 (continuation)

receptor	IHC-method	tumor 1		tumor 2		tumor 3		tumor 4		tumor 5		comments
		I	Q	I	Q	I	Q	I	Q	I	Q	
p-HER2	HOPE_nP_0CC2	0	0	0	0	0	0	0	0	0	0	
	HOPE_nP_8CC2	1	1_{focal}	1	1_{focal}†	1	1†	1	1_{focal}†	1	1_{focal}†	
	HOPE_nP_36CC2	1	1_{focal}	1	1†	1	1†	1	1†	1	1†	
	HOPE_nP_60CC2	1	1_{focal}	1	1†	1	1†	1	1†	1	1†	
	HOPE_nP_84CC2	0	0	1	1	1	1_{focal}	1	1†	0	0	
	isotype control	0	0	1	1‡	1	1_{focal}‡	1	1‡	1	1_{focal}‡	
	F_60CC1 (BenchMark)	2	2 (95 %)	2	2 (90 %)	2	2 (80 %)	2	2 (80 %)	2	2 (90 %)	medium staining
	F_nP_60CC1 (manual)	not performed										

† cytoplasmatic staining (unspecific) beside membrane staining
‡ cytoplasmatic staining (unspecific)

scoring: intensity (I): 0 = no staining; 1 = weaker than standard formalin; 2 = equivalent to standard formalin (BenchMark deparaffinization); 3 = stronger than standard formalin
quantity (Q): 0 = no cells stained (0% of cells); 1focal = less than 10% of cells stained; 2 = equivalent to standard formalin (BenchMark deparaffinization); 3 = more cells stained than in standard formalin; F = standard formalin fixed and paraffin embedded tissue; HOPE = tissue fixed with HOPE® and paraffin embedded; staining quantity for standard formalin expressed in %
numbers in title of IHC method = time of antigen retrieval expressed in minutes, nP = manual deparaffinization, CC1 = antigen retrieval buffer (Ventana Medical Systems)

E) fixation HOPE® - cell line OVCAR-5

receptor	IHC-method	tumor 1		tumor 2		tumor 3		tumor 4		tumor 5		comments
		I	Q	I	Q	I	Q	I	Q	I	Q	
IGF-1R	HOPE_nP_30CC1	2	1 *	2	1 *	2	1 *	2	1 *	2	1	* decreased morphology (shrunken cells)
	F_60CC1 (BenchMark)	2	2 (90 %)	2	2 (80 %)	2	2 (90 %)	2	2 (80 %)	2	2 (70 %)	weak staining
p-HER2	HOPE_nP_36CC2	not evaluable (decreased morphology)						0	0	0	0	
	F_60CC1 (BenchMark)	2	2 (70 %)	2	2 (70 %)	2	2 (90 %)	2	2 (70 %)	2	2 (70 %)	medium staining

E) fixation HOPE® - cell line NCI-H322M

receptor	IHC-method	tumor 1		tumor 2		tumor 3		tumor 4		tumor 5		comments
		I	Q	I	Q	I	Q	I	Q	I	Q	
IGF-1R	HOPE_nP_30CC1	2	1	2	1	2	1	3	1	2-3	1	
	F_60CC1 (BenchMark)	2	2 (95 %)	2	2 (95 %)	2	2 (95 %)	2	2 (90 %)	2	2 (95 %)	medium staining
p-HER2	HOPE_nP_36CC2	2	1	1-2	1	*		1	1	2	1	* not evaluable (decreased morphology)
	F_60CC1 (BenchMark)	2	2 (80 %)	2	2 (80 %)	2	2 (70 %)	2	2 (80 %)	2	2 (70 %)	medium - strong staining

scoring: intensity (I): 0 = no staining; 1 = weaker than standard formalin; 2 = equivalent to standard formalin (BenchMark deparaffinization); 3 = stronger than standard formalin
quantity (Q): 0 = no cells stained (0% of cells); 1focal = less than 10% of cells stained; 2 = equivalent to standard formalin (BenchMark deparaffinization); 3 = more cells stained than in standard formalin; F = standard formalin fixed and paraffin embedded tissue; HOPE = tissue fixed with HOPE® and paraffin embedded; staining quantity for standard formalin expressed in %
numbers in title of IHC method = time of antigen retrieval expressed in minutes, nP = manual deparaffinization, CC1 = antigen retrieval buffer (Ventana Medical Systems)
